# Sedation management and processed EEG-based solutions during venovenous extracorporeal membrane oxygenation: a narrative review of key challenges and potential benefits

**DOI:** 10.1007/s10047-025-01494-y

**Published:** 2025-03-08

**Authors:** Lajos Szentgyorgyi, Samuel Henry Howitt, Heather Iles-Smith, Bhuvaneswari Krishnamoorthy

**Affiliations:** 1 School of Health and Society, University of Salford, Mary Seacole Building, Frederick Road Campus, Broad St, Frederick Road Campus, Salford, M6 6PU UK; 2 Manchester University NHS Foundation Trust, Cardiothoracic Critical Care Unit, Wythenshawe Hospital, Southmoor Road, Manchester, M23 9LT UK; 3 Division of Cardiovascular Sciences, University of Manchester, 46 Grafton Street, Manchester, M13 9NT UK; 4 Centre for Clinical and Care Research, Northern Care Alliance NHS Foundation Trust, Stott Lane, Salford RoyalSalford, M6 8HD UK

**Keywords:** Extracorporeal membrane oxygenation, Sedation, Monitoring, Processed EEG, Critical care

## Abstract

Extracorporeal membrane oxygenation (ECMO) is an established technique for managing severe cardiorespiratory failure. However, it is invasive and requires profound analgo-sedation during initiation and often throughout the therapy. Managing sedation in venovenous (VV) ECMO patients is particularly challenging due to the impact of ECMO circuits on pharmacokinetics and specific patient requirements. This can lead to unpredictable sedative effects and require multiple drugs at higher doses. Additionally, sedation is usually managed with traditional scoring methods, which are subjective and invalid during neuromuscular blockade. These uncertainties may impact outcomes. Recent clinical practice increasingly focuses on reducing sedation to enable earlier physiotherapy and mobilisation, particularly in patients awaiting transplants or receiving mechanical circulatory support. In this context, processed electroencephalogram-based (pEEG) sedation monitoring might be promising, having shown benefits in general anaesthesia and intensive care. However, the technology has limitations, and its benefits in ECMO practice have yet to be formally evaluated. This review provides insights into the challenges of ECMO sedation, including pharmacokinetics, unique ECMO requirements, and the implications of inadequate sedation scores. Finally, it includes a brief overview of the practicality and limitations of pEEG monitoring during VV-ECMO, highlighting a significant research gap.

**Supplementary Information:**

The online version contains supplementary material available at 10.1007/s10047-025-01494-y.

## Introduction

The utilisation of venovenous extracorporeal oxygenation (VV-ECMO) to treat refractory respiratory failure in adults has steadily increased over the past two decades, reaching 51,137 registered cases in the European Life Support Organisation’s (ELSO) database by the end of January 2024 [1]. Management of sedation during VV-ECMO can be challenging, with different depths of sedation required at various stages of the patient’s treatment. The current ELSO guideline recommends deep sedation for the first 12–24 h with lighter sedation as the patient becomes more stable [2].

The pharmacodynamics of analgesic and sedative medications can differ among patients based on age, sex, disease progression, and other coexisting medical conditions. To achieve the desired level of sedation for VV-ECMO, it may be necessary to administer multiple sedative drugs. ECMO circuits impact the pharmacokinetics of each medication to varying degrees [3]. As a result, maintaining the appropriate depth of sedation throughout VV-ECMO can be a complex process.

While this review focuses on VV-ECMO, allowing for a more in-depth discussion of the associated challenges and offering practical insights tailored to VV-ECMO management, there is a substantial crossover of considerations for VV and VA-ECMO. Pharmacokinetic considerations and the potential use of processed electroencephalogram (pEEG) monitoring apply to both treatments. Still, the sedation goals usually differ due to the distinct patient populations, clinical scenarios and sedation targets involved.

Depth of sedation is traditionally monitored using observer-dependent traditional scoring methods based on clinical assessments [4–6]. However, the Neurocritical Care Society's recent international expert consensus panel strongly recommends processed electroencephalogram (pEEG) sedation monitoring for ECMO patients under neuromuscular blockade [7]. While traditional EEG methods are often complex and impractical for routine clinical use, requiring specialised knowledge to interpret data, pEEG monitoring is easier, independent from observer bias and can continuously represent and objectively assess the pharmacodynamics of sedative agents. The bispectral index (BIS) device is the most widely used and extensively studied EEG-based monitor in clinical practice [8]. It has also received a recommendation from the Society of Critical Care Medicine (SCCM), which was published in the Pain, Agitation/Sedation, Delirium, Immobility (rehabilitation/mobilisation), and Sleep (disruption) (PADIS) guideline [9].

Evidence regarding pEEG sedation monitoring during general anaesthesia and intensive care sedation is growing [7, 10]. This review aims to answer the question of whether processed EEG monitoring during VV-ECMO could offer benefits when considering and exploring various challenges of ECMO sedation. The objectives are to examine the literature to find potential advantages and disadvantages of pEEG sedation monitoring during VV-ECMO treatment and to provide an overview of current sedation practices in ECMO. The review aims to explore the role of pEEG in optimising sedation strategies, addressing the limitations of traditional sedation scoring systems, and evaluating the clinical evidence supporting its capacity to enhance patient outcomes through objective, real-time assessments of sedation depth. Furthermore, it seeks to identify gaps in current research, propose directions for future studies, and discuss practical considerations for integrating pEEG into ECMO management protocols. This review hopes to lay the groundwork for evidence-based practices and adopt innovations in ECMO care by synthesising existing knowledge.

## Methods

A literature search was conducted using multiple databases, including Medline (Medical Literature Analysis and Retrieval System Online by U.S. National Library of Medicine), EMBASE (Excerpta Medica dataBASE), CINAHL (Cumulative Index to Nursing and Allied Health Literature), the Cochrane Library (https://www.cochranelibrary.com/) and Web of Science. The literature search aimed to explore the potential practicality, limitations, advantages, and disadvantages of using processed EEG for sedation monitoring in intensive care settings and to understand the challenges of VV-ECMO sedation. The following terms and Boolean operators were searched in the title and abstract fields: “extracorporeal” or “ECMO” or “Extracorporeal Membrane Oxygenation” and “BIS” or “bispectral index” or “pEEG” or “processed electroencephalogra*” or “processed EEG”. A second search included “extracorporeal” or “ECMO” or “Extracorporeal Membrane Oxygenation” and “Narcotrend” or “entropy” or “SedLine” to find other processed EEG monitors in ECMO practice. This structured search could not identify any relevant papers that met the criteria for a systematic review regarding using pEEG sedation monitoring in the VV-ECMO population. Therefore, subsequent searches were conducted to comprehensively explore the applicability and implications of pEEG sedation monitoring in this specific context. These extended searches included terms and Boolean operators such as “extracorporeal” or “ECMO” or “Extracorporeal Membrane Oxygenation” combined with the term “sedat*.” Furthermore, we investigated trends in processed EEG monitoring in critical care settings using terms like “BIS” or “bispectral index” or “pEEG” or “processed electroencephalography” or “processed EEG,” along with terms such as “intensive care” or “critical care” or “ventil*”. The search criteria did not include any restrictions on publication date. The references of the identified papers were thoroughly screened, and relevant literature was included in the analysis.

Due to the lack of specific studies investigating pEEG sedation monitoring in VV-ECMO, conducting a systematic or scoping review to summarise the literature would be inappropriate. Instead, we opted for a narrative review to comprehensively map the relevant literature and identify key factors related to pEEG sedation monitoring in VV-ECMO. In approaching the topic, we considered two major themes: sedation during VV-ECMO treatment and the potential role of pEEG monitoring in VV-ECMO care.

## Theme 1: sedation during VV-ECMO treatment

Sedation and analgosedation (administration of analgesic agents with significant sedative effects) are integral components of patient care in ECMO settings. The aim is to ensure patients are comfortable, pain-free, and experience minimal anxiety while maintaining a safe and manageable environment, thereby likely avoiding delirium. These objectives are in line with the principles outlined in the 2018 PADIS guideline [9] and with the eCASH concept (early Comfort using Analgesia, minimal Sedatives and maximal Humane care) proposed by Vincent et al. in 2016 for general intensive care [11].

### Considerations about deep sedation

During the initial phase of VV-ECMO, patients are, by definition, in established organ failure and are often very unstable. Therefore, early light sedation goals may not apply to patients receiving VV-ECMO. Most VV-ECMO runs are conducted in patients with ARDS when respiratory volumes are usually minimal and decrease further in the early phase of VV-ECMO treatment. The secretion burden is typically significant, with frequent bronchospasm, and there may be considerable airway bleeding. Under such conditions, the potential respiratory benefits of light sedation are unlikely to be realised, and lighter sedation may not be achievable. Moreover, despite conflicting results in the literature [12–14], these patients are also often treated in the prone position and require substantial cardiovascular support to manage systemic vasodilation, pulmonary vasoconstriction, or right heart strain. It, therefore, remains common to manage the early phase of ECMO treatment with heavy sedation [5, 6]. This traditional tendency towards heavy sedation may have also resulted from concerns that lightly sedated patients were more likely to move and dislodge cannulae or breathe against the ventilator, increasing their intrathoracic pressure and causing issues with ECMO circuit flow. Fortunately, experience has shown that these fears are largely unfounded. Indeed, there is a growing trend to reduce sedation as soon as the patient’s condition allows and ultimately wean sedation entirely while still delivering VV-ECMO in some instances [15, 16]. These recent developments have concerned moving away from the traditional use of heavy sedation, which may be throughout the entire ECMO treatment; however, during the first few days or occasionally weeks, patients are profoundly sedated [5, 17].

### Considerations about light sedation

Recent studies in the general critical care cohort have shown that unnecessarily deep sedation is associated with an increase in unpleasant delusional memories, nightmares, hallucinations, morbidity and mortality [18–22]. Therefore, as soon as patients are stable enough, the recommendation in general critical care is to achieve light-level analgosedation with daily sedative interruptions [2, 9]. It should be noted that there is no universal definition for deep or light-level sedation; however, a score of −3 or below on the widely used Richmond Agitation-Sedation Scale (RASS) is usually considered deep or profound sedation. (A brief description of the three most commonly used sedation scores is found in the supplementary material number 1). There have also been some dissenting studies regarding the benefits of daily sedation holds [23]. While evidence in the ECMO population is lacking, some ECMO-specific considerations must be considered; moving towards lighter sedation in modern ECMO care seems beneficial [24]. Indeed, it has been shown that awake ECMO might be advantageous in specific patient populations, particularly in the “bridge-to-transplant” scenario [25–27], when planned and semi-elective VV-ECMO support is initiated [28–36].

A recent systematic review by Belletti et al. [37] suggests that awake ECMO is a feasible option, particularly in the bridge-to-lung transplantation scenario, resulting in a low expected failure rate and common complications such as delirium, agitation, worsening respiratory failure, and bleeding. However, further studies need to establish optimal timing, impact on survival and selection criteria [37]. There have also been reports of selected cases and case series where awake VV-ECMO was used for patients with acute respiratory distress syndrome (ARDS) [38–45]. Galante et al. reported a series of 25 awake VV-ECMO cases when 6.8% of 365 COVID-19 patients were treated as awake, resulting in shorter ECMO runs and an overall survival rate of 76% [45]. VA-ECMO or other extracorporeal mechanical circulatory support might also be suitable for awake extracorporeal support [46]. VA-ECMO duration is typically shorter, and patients can be extubated early after mechanical support and gradually taken off sedation, potentially avoiding delirium [47]. This approach helps them remain alert throughout their treatment, enabling ongoing verbal communication, oral intake of nutrients, and participation in physiotherapy sessions to prevent muscle atrophy [48–50]. Various cannulation techniques and unique ECMO configurations facilitate mobilisation and awake physical and psychological rehabilitation [16, 51–56], preserving the beneficial effect of spontaneous breathing on respiratory mechanics [57].

### Considerations about the awake approach

Although the awake approach may yield morbidity and mortality benefits [37, 58], it is essential to acknowledge the potential complications associated with this approach. Maintaining patient compliance with therapy is crucial in awake ECMO. A case study by Haneke et al. highlights the risk of accidental device removal in awake patients, which can have devastating consequences. Although the presented patient survived an accidental decannulation, in similar cases, there is a risk of severe exsanguination or death due to the cessation of essential extracorporeal life support [39]. Similarly, mobility during ECMO treatment can lead to a sudden reduction in ECMO flow or bleeding issues [59]. Furthermore, anxiety, agitation due to inadequate analgosedation, or delirium can contribute to hyperventilation with self-inflicted lung injury, potentially resulting in long-term psychological and physiological consequences [33, 60]. Timofte et al. reported an unconventional yet seemingly effective approach to managing high respiratory drive and associated hyperventilation, known as "drowning syndrome” in VV-ECMO. They administered neuromuscular blocking agents, leading to adaptive periodic paralysis, also requiring some degree of sedation [61]. It is important to note that awake patients may require occasional profound sedation for tracheostomies and bronchoscopies for airway clearance, even though they are mostly awake while receiving ECMO therapies [62].

pEEG monitoring could potentially guide the initial or subsequent phases of deep sedation and might benefit the following phase of controlled sedation lightening.

### Challenges of VV-ECMO sedation

The challenges associated with VV-ECMO sedation can be attributed to the challenges related to patients’ physiology, requirements specific to VV-ECMO, pharmacokinetic effects of ECMO circuits and the shortfalling role of traditional sedation scores.

#### Considerations related to abnormal physiology

ECMO patients exhibit dysfunction of other organs in addition to their respiratory failure. Their renal function is often compromised, and renal replacement therapy is not uncommon. There may be liver impairment, and gastrointestinal absorption is frequently suboptimal. These factors can affect the absorption, metabolism and excretion of sedative medications. Similarly, sepsis is often a complicating factor and may result in pyrexia, hyperdynamic circulation, hypoalbuminemia, capillary leak, and elevated levels of inflammatory proteins. These factors also significantly impact the pharmacokinetics of sedative drugs. It is worth noting, however, that these features are not exclusive to VV-ECMO and are commonly observed in general critical care settings [63].

#### Considerations specific to VV-ECMO care

While most VV-ECMO circuit configurations can allow patient movement without negatively impacting gas exchange [64], there are specific situations where sudden drops in blood flow through the circuit can result in rapid oxygen desaturation [65]. Although such issues can often be managed with fluid administration [66], there are occasions, particularly in the presence of elevated intrathoracic or intraabdominal pressures or prone positions, where deep sedation and muscle relaxation become necessary.

VV-ECMO patients may also require frequent and potentially painful procedures such as bronchoscopies, line or drain insertions, and tracheostomies. Often, these procedures occur under anaesthesia, and these periods of profound sedation may combine, resulting in prolonged periods of deeper sedation. Similarly, if patients cannot be managed safely with lighter sedation, heavier sedation may be required to prevent the accidental removal of a life-saving device.

Although lightening or cessation of sedation may become feasible as the course progresses to facilitate patient mobilisation [4, 17, 67, 68], deep sedation in the early phase of VV-ECMO requires considerably higher doses of sedatives than in the case of non-ECMO patients, frequently requiring a combination of multiple sedatives [69–71]. Therefore, continuous sedation monitoring could be essential when managing multiple agents to achieve sedation targets.

#### Pharmacokinetic considerations

ECMO significantly influences the pharmacokinetics of sedative drugs, exacerbating sedation challenges [3, 67, 72–77]. Ex vivo studies investigating the various sedative agents in ECMO circuits have shown sequestration of lipophilic and protein-bound sedatives, such as midazolam, propofol, dexmedetomidine and fentanyl and its derivatives, which may suffer significant loss within the ECMO circuits [78–91]. These studies used different circuits with varying materials, coatings, and drugs for various durations. Therefore, summarising the studies here is impractical and is not the focus of this review. Nevertheless, it must be noted that significant drug loss was consistently observed. This phenomenon might explain the requirement for higher doses of lipophilic sedatives like fentanyl derivatives and midazolam in clinical practice [3, 67, 72–76]. In contrast, hydrophilic morphine demonstrates less sequestration. Protein deposits in the oxygenators can also contribute to the trapping of protein-bound agents [90]. Dexmedetomidine suffers at least 50% loss in vitro ECMO circuits [83], and Dallefeld et al. [86] concluded that this loss is due to extraction by the oxygenator [86].

Extracorporeal circulation, as an extension of the human circulatory system, induces haemodilution, leading to an increased volume of distribution and, subsequently, lower plasma concentrations of drugs. Large ECMO cannulas in the inferior vena cava may contribute to liver and kidney congestion. Furthermore, ECMO may adversely affect hepatic cytochrome pathways. However, additional research is needed to understand this impact fully [72, 75, 76].

While pharmacokinetic research provides valuable insights into understanding the factors influencing sedation, it is essential to note that pharmacokinetic studies are often of limited assistance in guiding sedation practices [92]. pEEG monitoring could allow the sedative effect of medications to be formally quantified, facilitating the titration of medications and circumnavigating some of the pharmacological and physiological complexities.

##### Volatile sedation

Several studies have assessed the potential benefits of volatile sedation with sevoflurane or isoflurane inhalation during ECMO support [93–97]. However, these studies have limitations, such as small sample sizes and heterogeneity in patient populations [95]. The opioid-sparing effect, swift recovery from sedation, and the opportunity for early neurological assessments following volatile sedation may present advantages akin to those observed in general critical care [98, 99]. Nevertheless, the studies concluded that volatile sedation is feasible but does not significantly affect primary and secondary outcomes such as mortality, length of stay, ECMO duration, or delirium [96, 97]. The reliability of volatile agents' minimum alveolar concentration (MAC value) as a reliable indicator of sedation depth is uncertain due to the altered respiratory mechanics of patients receiving ECMO. Moreover, despite advancements in scavenging systems, the inherent risks of occupational exposure and the associated environmental impact (such as the greenhouse gas effect) may attenuate the attractiveness of this approach [100, 101].

#### Role of sedation scores

Surveys conducted by Buscher et al. in 2013, Marhong et al. in 2017 and Dzierba et al. in 2019 have shown that sedation practices during ECMO care vary considerably worldwide [4–6]. However, most departments use the same observer-dependent scoring systems for sedation monitoring in ECMO as in other ICU patients. These tools include the Richmond Agitation Sedation Scale (RASS), Ramsay Sedation Scale (RSS), Riker Sedation-Agitation Scale (SAS), and, less frequently, other scales [4–6]. (See supplementary material 1).

These sedation scores are subjective and not capable of continuous real-time monitoring. They provide isolated snapshot values for assessing agitation and response to stimulation rather than the depth of sedation per se. Moreover, the reactions required for their use might be prevented by neuromuscular blockade.

Sedation scores may result in oversedation, as evidenced by a recent study by Favre et al. on general ICU patients [102]. In this study, the RASS score between − 5 and − 4 was targeted while treating clinicians were blinded to pEEG data. pEEG monitoring in the form of Patient State Index (PSI) by the SedLine® monitor (Masimo, Irvine, CA, USA) was found to be below 25 (indicating EEG suppression) for half of the total monitored time. A low PSI was associated with delirium, potentially negatively influencing the duration of mechanical ventilation and lengthening the ICU stay [102].

pEEG monitoring could allow reliable, real-time and continuous assessment of sedation depth in VV-ECMO care. However, no published studies have analysed the benefits of pEEG for sedated patients receiving ECMO.

**Fig. 1:**
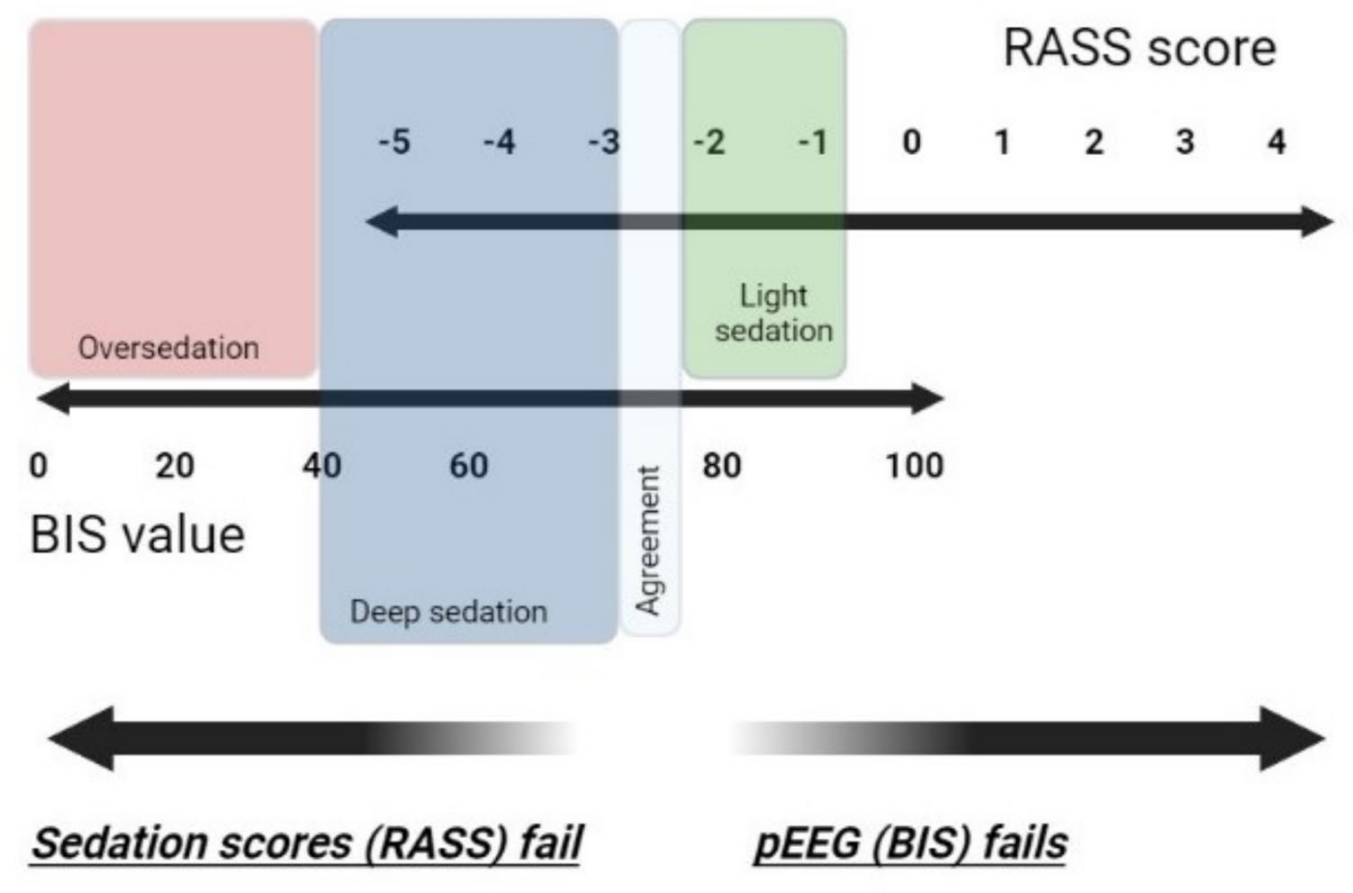
Relationship between the Richmond Agitation Sedation Scale (RASS) and the Bispectral Index (BIS) in the context of sedation depth and monitoring. (In light sedation, BIS monitoring is impractical due to motion artefacts and the ease of verbal assessments and patient communication. BIS monitoring is also ineffective for assessing agitation. In deep sedation, sedation scoring becomes more subjective; spontaneous movements might be misinterpreted as inadequate sedation depth, often leading to increased sedative doses. When patients reach an RASS score of − 5, and often at − 4, only pEEG monitoring can detect oversedation due to excessive sedative administration. Additionally, all scoring methods employ physical stimuli that may inevitably disrupt sleep patterns, temporarily decreasing the depth of sedation.)

Figure 1 illustrates the relationship between the widely used sedation score, RASS, and the most popular processed EEG monitor, Bispectral Index (BIS), in the context of sedation depth and monitoring.

## Theme 2: potential role of pEEG monitoring in VV-ECMO care

pEEG-based sedation monitoring is most reliable in deep sedation [103]. A recent general critical care study conducted by Idei et al. concluded that pEEG parameters, specifically the Patient State Index (PSI) measured by the SedLine® (Masimo, Irvine, CA, USA), can accurately distinguish between deep and light sedation with high sensitivity and specificity, and correlate well with RASS measurements [104]. pEEG monitoring allows for objective and continuous sedation assessment, which can help prevent inappropriate light or profound sedation in ICU patients [104]. The National Audit Project 5 (NAP5) audit in the United Kingdom highlighted the issue of intraoperative awareness during total intravenous anaesthesia in operating theatres and mandated depth of anaesthesia monitoring during surgical procedures [105]. Based on this reasoning, it would be rational to recommend monitoring sedation levels using pEEG monitors, particularly when ECMO patients undergo interventions, transfers, or procedures where awareness is unwanted and more likely.

The usefulness of pEEG monitoring outside deep sedation is unclear, and the effects on required sedative drug doses in critical care patients have not been convincingly demonstrated. In Bass et al.’s small retrospective cohort study, no significant differences in sedation or analgesia requirements were found between BIS-guided and RASS-guided intensive care patients ventilated for ARDS [106]. However, in this study, almost all BIS patients (99%) required deep sedation (BIS < 60), while nearly half of the RASS-guided cases needed only light sedation (RASS ≥ − 3), which may make the comparison biased [106]. In a smaller randomised control trial that included only 50 lightly sedated (BIS > 70) patients from a very heterogeneous ICU cohort, Weatherburn et al. found no difference in sedative drug requirements when pEEG was used [107]. However, these results should be interpreted with caution because of the small number of patients and the heterogeneity of their cohort. In contrast, Olson et al. reported that pEEG monitoring was associated with receiving reduced doses of propofol and opioids in lightly sedated (RASS − 2) general critical care patients [108]. In their prospective randomised trial that included 300 patients, there was, however, an unexpected increase in the doses of sedatives required if dexmedetomidine and benzodiazepines were chosen as sedatives. The study found no significant improvement in clinical outcomes associated with pEEG monitoring [108]. This study corresponded well with the authors’ previous study involving 67 neurocritical care patients [109]. In a recent randomised controlled trial, Huespe et al. found that BIS-guided sedation significantly reduced the sedative doses in deeply sedated critical care patients while maintaining higher levels of EEG activity [110]. However, BIS guidance did not improve delirium-free and coma-free days overall, except in a subgroup of patients with deep sedation lasting more than 24 h, in which the BIS-guided group experienced significantly more delirium-free and coma-free days (median, 1 day [IQR, 0–9 days] vs median, 8 days [IQR, 0–13 days]; P ¼ 0.04). The target BIS range in the BIS-monitored arm was 40–60, while the target RASS score in the clinical assessment group was between − 4 and − 5 [110]. Notably, the improved subgroup closely resembles the ECMO population.

A systematic review by Shetty et al. found insufficient evidence to draw conclusions regarding the effects of BIS monitoring for sedation on clinical outcomes or resource utilisation in critically ill mechanically ventilated adults [111]. However, it is essential to note that no specific research has been conducted to explore the potential sedative-sparing effects of pEEG monitoring during ECMO therapy.

Clinicians and nurses caring for patients receiving ECMO must divide their attention among multiple tasks [112, 113]. The cognitive load is high, and a protocolised approach to titration of sedatives based on real-time parameters, such as the pEEG parameters, may alleviate some of this burden and reduce nursing workload by saving time otherwise spent assessing sedation scores hourly. While it is crucial to maintain documented sedation score targets, adjusting sedative doses based on pEEG parameters may require less frequent scoring.

In the future, pEEG-guided closed-loop sedative administration systems could be integrated into clinical practice, reducing the need for human intervention in sedation administration. However, it is essential to note that these systems are still in the developmental stage. They are not currently available for patient care and have not been designed for ECMO care [114, 115]. (The features of the most commonly used pEEG sedation monitors are summarised in supplementary material number 2.)

### Potential advantages of using pEEG monitoring in ECMO

Acute neurological complications, such as intracranial haemorrhages, ischaemic strokes, and seizures, occur in approximately 10% of the VV-ECMO and 15% of VA-ECMO populations, respectively [116]. Such complications may result in sudden changes in pEEG values, prompting further tests to determine the diagnosis [117, 118]. However, it is also essential to recognise that the effects of neurological injuries on regional blood flow, often in the frontal region, may reduce the sensitivity and utility of available pEEG monitors in assessing sedation depth [119]. Peluso et al. observed that 38% of ECMO patients with severe EEG background abnormalities were significantly associated with adverse neurological outcomes and mortality [120]. On the other hand, studies suggest that in those patients who suffer prolonged hypoxia or circulatory arrest, there is potential to use pEEG for neuro-prognostication [121–124]. Combining pEEG with near-infrared spectroscopy (NIRS) monitoring in VV-ECMO patients might optimise sedation depth by aligning it with brain activity and cerebral oxygenation demand [125].

A significant proportion of VV-ECMO patients (60–98%) may experience delirium [126, 127]. Also, oversedation in critical care has been associated with an increased risk of delirium [21, 128]. However, evidence regarding the benefit of pEEG monitoring to prevent delirium is conflicting. Nevertheless, a recent systematic review and meta-analysis by Sumner et al. suggested that pEEG monitoring may help prevent excessive intraoperative anaesthesia, potentially reducing postoperative delirium incidence [129]. However, the included trials and their statistical and clinical methods were very heterogeneous, and the primary result of this meta-analysis did not show a statistically significant benefit [129]. Despite some methodological concerns, the included ENGAGES (Electroencephalography Guidance of Anesthesia to Alleviate Geriatric Syndromes) trial in 2019 did not support pEEG monitoring to reduce delirium [130, 131]. Also, the recent ENGAGES—Canada large prospective multicentre randomised trial has failed to demonstrate that pEEG decreases delirium in an elderly cardiac surgical population [132]. Research on the ECMO population is lacking.

Light sedation might also worsen delirium. A recent retrospective registry study by Sun et al. found an increased delirium incidence in lightly sedated adult patients receiving VV-ECMO for severe COVID-19 ARDS. The authors hypothesised that inadequate light sedation might have exacerbated pain and discomfort, leading to patient-ventilator dyssynchrony and, ultimately, adverse delirium incidence [126].

Jarry et al. demonstrated that pEEG-guided anaesthesia in cardiac surgeries with extracorporeal cardiopulmonary bypass resulted in reduced inotropic and vasoactive drug doses upon ICU arrival, lower anaesthetic and opioid requirements during surgery, decreased central venous pressures, reduced fluid needs and intraoperative bleeding, and a shorter duration of mechanical ventilation [133]. While extrapolation from other studies and different patient populations can lead to false conclusions, it may also highlight trends and lacking evidence. Similar benefits of pEEG-guided sedation during VV-ECMO have yet to be investigated.

pEEG monitoring in sedated VV-ECMO patients could prevent unnecessarily profound and inadequate light sedation. However, further research is needed to determine if this approach can reduce the incidence of delirium, lower drug doses, or lead to more favourable physiological parameters in the VV-ECMO population.

### Possible barriers to pEEG monitoring in VV-ECMO sedation

pEEG monitors are designed to measure electrical signals from the frontal brain structures that may not represent the accurate anatomical structures contributing to consciousness. These signals can be susceptible to interference from external sources. One common type of interference is electromyographic (EMG) artefacts, which can arise from non-paralysed patients who are shivering or moving. Interestingly, EMG signals may be necessary to derive accurate BIS numbers, as suggested by a study conducted on healthy volunteers by Schuller et al. [134]. This finding has important implications for patients under neuromuscular blockade. In such cases, deeper pEEG numbers may be necessary to ensure that patients are adequately sedated and to prevent the risk of recollections and inadvertent awareness. However, high muscular activity in the intensive care unit can lead to an overestimation of BIS, resulting in falsely higher readings that may eventually cause BIS-induced oversedation [135]. In addition, pEEG sedation monitoring may become less accurate as age increases [136]. Additionally, sources of electrical interference, such as warming blankets, pneumatic mattresses, and mechanical and ECMO pumps, may also affect the accuracy of EEG readings [137]. pEEG devices from various manufacturers may also show different tendencies despite processing the same EEG signal, as was recently published by Hight et al. [138].

Ketamine as an adjunct in ECMO analgosedation may offer several benefits with minimal drawbacks [139–144]. However, it does not necessarily reduce sedatives or opioids [141]. Unfortunately, the administration of ketamine can lead to an artificial increase in the commonly used BIS numbers. This effect can be attributed to the drug’s ability to elevate the cerebral metabolic rate or increase theta activity within the EEG power spectrum [145, 146]. Therefore, when ketamine is used, it is crucial to interpret pEEG values cautiously and consider alternative monitoring methods, such as sedation scores, to assess sedation levels accurately. As mentioned above, Olson et al. reported that pEEG monitoring increased dexmedetomidine and benzodiazepine requirements, which might be attributed to the various electrophysiological effects of these agents, besides the light sedation targets and perhaps the anti-anxiety and anti-delirium effects of these medications [108].

Noxious stimuli may also affect pEEG parameters. However, at best, this surrogate is challenging to assess reliably, and more effective nociception monitors are available [147, 148]. Critical care staff must also be aware that targeting pEEG levels in ECMO sedation will not necessarily guarantee immobility, which might occasionally be necessary.

**Fig. 2:**
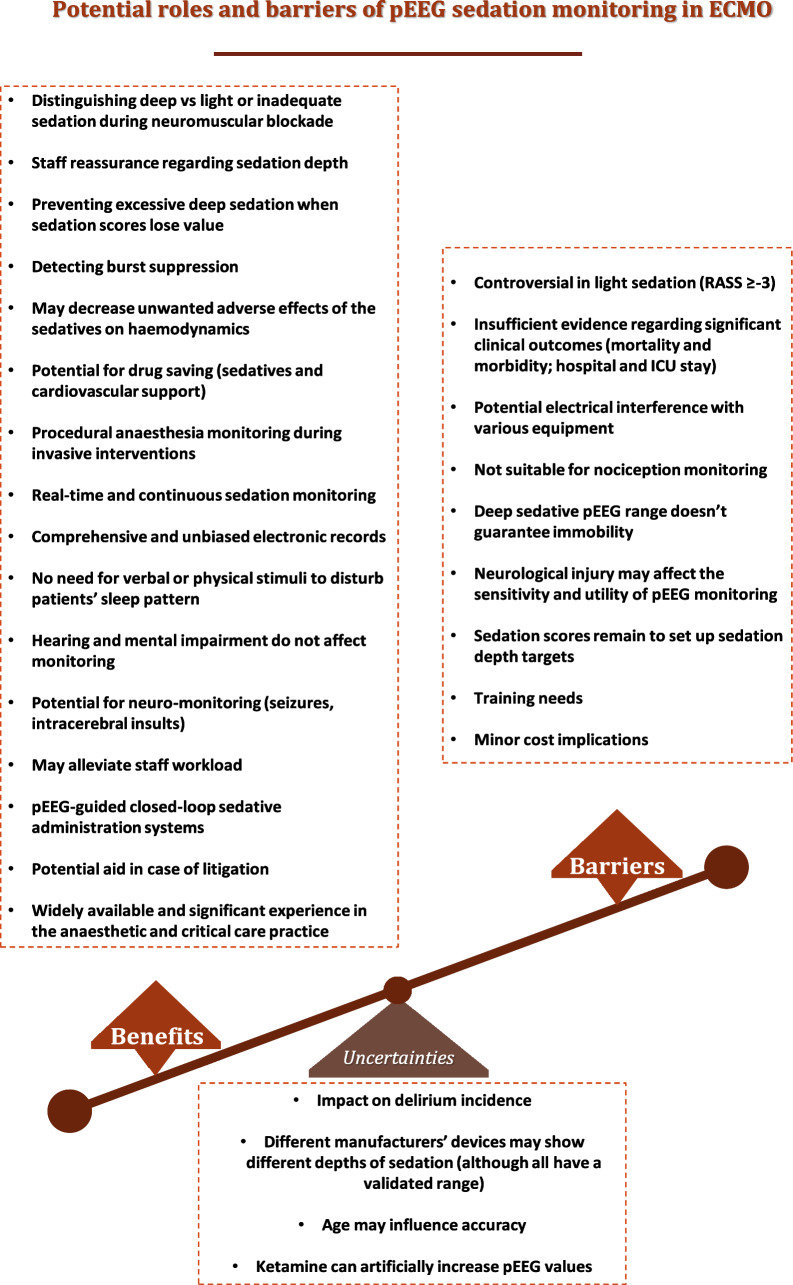
Potential roles and barriers of pEEG sedation monitoring in ECMO. The benefits and barriers of pEEG sedation monitoring in VV-ECMO may differ in their impact, with some aspects being more significant than others

Figure 2 summarises the potential advantages, barriers and uncertainties regarding pEEG sedation monitoring in VV-ECMO care.

## Strengths and limitations

The strengths of this narrative review include the substantial literature search and a comprehensive approach to mapping evidence, as well as the identification of knowledge gaps, theories, and key factors related to the concept of VV-ECMO sedation and pEEG sedation monitoring in VV-ECMO care. While this review primarily addresses VV-ECMO sedation, it also includes relevant details from studies related to general ICU and VA-ECMO populations when discussing transferrable concepts to ensure a comprehensive overview. The limitations are the lack of specific studies investigating the pEEG monitoring during VV-ECMO and the wide variety of literature providing information about ECMO and pEEG sedation monitoring separately.

## Conclusion

Sedation during VV-ECMO therapy poses several challenges. VV-ECMO alters the pharmacokinetics of sedatives, and the unique needs of this patient population make it difficult to accurately assess, conduct and monitor sedation using traditional scoring methods. Although pEEG monitoring is gradually becoming a standard clinical practice in general critical care, there is no established evidence for its use in VV-ECMO therapy. Despite the lack of hard evidence, using pEEG for sedation monitoring in ECMO care has been advocated and supported in the literature [7, 113, 149–151]. Given its proven utility in other populations and the absence of significant contraindications, the authors believe pEEG monitoring should be routinely implemented in all ECMO cases. However, while it has never been considered ineffective or unsafe, nationally endorsed ECMO guidelines in the United Kingdom have yet to recommend pEEG-based sedation monitoring specifically for ECMO care.

Therefore, there is a clear need for further research to clarify the advantages and limitations of pEEG monitoring in this setting. Future research should aim to establish optimal and safe pEEG targets for ECMO sedation, develop protocols that minimise the use of sedatives and opioids facilitating sedation weaning, and correlate specific pEEG changes with neurological outcomes and age-related changes. Integrating pEEG with other monitoring methods, such as near-infrared spectroscopy, may be particularly beneficial. In the future, machine learning or artificial intelligence-based models that synthesise multiple physiological signals (e.g., pEEG, ECMO parameters, near-infrared spectroscopy, vital signs, and blood gas data) might have the potential to improve outcome predictions. Finally, additional studies are needed to evaluate the cost-effectiveness of pEEG and its impact on clinical workflows in resource-intensive ECMO settings.

## Supplementary Information

Below is the link to the electronic supplementary material.

Supplementary file1 (DOC 65 KB)

Supplementary file2 (DOC 56 KB)

## Data Availability

This article is a narrative review and does not involve generating or analysing original research data. All information presented in this article is derived from publicly available literature sources (described in the methods section), which are appropriately cited in the reference list. Therefore, a data availability statement is not applicable.
