# Supplementary material for: Sedation management and processed EEG-based solutions during venovenous extracorporeal membrane oxygenation: a narrative review of key challenges and potential benefits
Source: J Artif Organs. 2025 Mar 8;28(3):293–307. doi: 10.1007/s10047-025-01494-y (PMC12373690; doi:10.1007/s10047-025-01494-y)
Supplement: Supplementary file 1 — Supplementary file1 (DOC 65 KB) [file 10047_2025_1494_MOESM1_ESM.doc]

# Supplementary material 1. – Sedation Scores

| **Richmond Agitation-Sedation Scale (RASS)[1]** | | | **Ramsay Sedation Scale (RSS)[2]** | | **Riker Sedation-Agitation Scale (SAS)[3]** | | |
| --- | --- | --- | --- | --- | --- | --- | --- |
| *Score* | *Term* | *Description* | *Score* | *Description* | *Score* | *Term* | *Description* |
| **+4** | Combative | Overtly combative or violent, immediate danger to staff | **1** | Anxious and agitated or restless or both | **7** | Dangerous agitation | Pulling at ET tube, trying to remove catheters, climbing over bed rail, striking at staff, thrashing side-to-side |
| **+3** | Very agitated | Pulls on or removes tube(s) or catheter(s) or exhibits aggressive behavior toward staff |  |  | **6** | Very agitated | Does not calm, despite frequent verbal reminding of limits; requires physical restraints, biting ET tube |
| **+2** | Agitated | Frequent nonpurposeful movement or patient-ventilator dys-synchrony |  |  | **5** | Agitated | Anxious or mildly agitated, attempting to sit up, calms down to verbal instructions |
| **+1** | Restless | Anxious or apprehensive but movements not aggressive or vigorous |  |  |  |  |  |
| **0** | Alert and calm |  | **2** | Cooperative, oriented, and tranquil | **4** | Calm and cooperative | Calm, awakens easily, follows commands |
| **-1** | Drowsy | Not fully alert, but has sustained (>10 seconds) awakening, with eye contact, to voice | **3** | Responds to commands only |  |  |  |
| **-2** | Light sedation | Briefly (<10 seconds) awakens with eye contact to voice |  |  | **3** | Sedated | Difficult to arouse, awakens to verbal stimuli or gentle shaking but drifts off again, follows simple commands |
| **-3** | Moderate sedation | Any movement (but no eye contact) to voice | **4** | Brisk response to a light glabellar tap or loud auditory stimulus | **2** | Very sedated | Arouses to physical stimuli but does not communicate or follow commands, may move spontaneously |
| **-4** | Deep sedation | No response to voice, but any movement to physical stimulation | **5** | Sluggish response to a light glabellar tap or loud auditory stimulus | **1** | Unarousable | Minimal or response to noxious stimuli, does not communicate or follow commands |
| **-5** | Unarousable | No response to voice or physical stimulation | **6** | No response to a light glabellar tap or loud auditory stimulus |  |  |  |
| Performed using a series of steps: observation of behaviors (score +4 to 0), followed (if necessary) by assessment of response to voice (score -1 to -3), followed (if necessary) by assessment of response to physical stimulation such as shaking shoulder and then rubbing sternum if no response to shaking shoulder (score -4 to -5).[1] | | | Performed using a series of steps: observation of behavior (score 1 or 2), followed (if necessary) by assessment of response to voice (score 3), followed (if necessary) by assessment of response to loud auditory stimulus or light glabellar tap (score 4 to 6).[2] | | Performed using a series of steps: observation of behavior (score 7 to 5), followed (if necessary) by assessment of response to voice (score 4), followed (if necessary) by assessment of response to voice or gentle shaking (score 3), followed (if necessary) by physical stimuli (score 2), followed (if necessary) by noxious stimuli (score 1).[3] | | |

Table 1. Comparison of the most commonly used sedation scores in critical care. (It should be noted that all use verbal or physical stimuli to assess sedation and a snapshot response.)

## *References to supplementary material 1.*

1. Sessler CN, Gosnell MS, Grap MJ, Brophy GM, O'Neal PV, Keane KA, et al. The Richmond Agitation-Sedation Scale: validity and reliability in adult intensive care unit patients. Am J Respir Crit Care Med. 2002;166(10):1338-44. doi: 10.1164/rccm.2107138.

2. Sessler CN, Grap MJ, Ramsay MA. Evaluating and monitoring analgesia and sedation in the intensive care unit. Crit Care. 2008;12 Suppl 3(Suppl 3):S2. doi: 10.1186/cc6148.

3. Riker RR, Picard JT, Fraser GL. Prospective evaluation of the Sedation-Agitation Scale for adult critically ill patients. Critical Care Medicine. 1999;27(7):1325-9.
