# Supplementary material for: Sedation management and processed EEG-based solutions during venovenous extracorporeal membrane oxygenation: a narrative review of key challenges and potential benefits
Source: J Artif Organs. 2025 Mar 8;28(3):293–307. doi: 10.1007/s10047-025-01494-y (PMC12373690; doi:10.1007/s10047-025-01494-y)
Supplement: Supplementary file 2 — Supplementary file2 (DOC 56 KB) [file 10047_2025_1494_MOESM2_ESM.doc]

# Supplementary material 2. – processed EEG monitors

|  | **BIS™[1]** | **SedLine®[2]** | **Entropy®[3]** | **Narcotrend®[4, 5]** | |
| --- | --- | --- | --- | --- | --- |
| **Manufacturers** | **Medtronic Plc/Covidien Plc** | **Masimo Corporation** | **GE HealthCare Technologies, Inc.** | **Narcotrend Group** | |
| *Products’ webpage* | *https://www.medtronic.com/covidien/en-us/products/brain-monitoring/bis-monitoring-system.html* | *https://professional.masimo.co.uk/products/continuous/root/root-sedline/#Schedule%20an%20Evaluation* | *https://clinicalview.gehealthcare.com/quick-guide/entropy-monitoring-valuable-tool-guiding-delivery-anesthesia* | *https://narcotrend.de/home-en.html* | |
| **Displayed parameters** | - **Bispectral Index (BIS)**   **numeric values 0-100**   - BIS™ trend over time chart - EEG waveform (1 frontal waveform) - Density Spectral Array (DSA): spectrograms representing the power of the EEG frequencies. - Spectral Edge Frequency (SEF) (Hz)   Median Frequency (MF) (Hz)   - Burst suppression ratio (SR)   (0-100%)   - Burst suppression time or count number   (burst/minutes or ST: min.)   - Electromyograph (EMG) signal indicator   (single bar chart)   - Signal quality indicator   (bar chart)   - Signal quality index (SQI) (calculated) | - **Patient State Index (PSI)**   **numeric values 0-100**   - EEG waveforms (bilateral 4 frontal waveforms) - Density Spectral Array (DSA): spectrograms representing the power of the EEG frequencies. - SEFL/SEFR (Spectral Edge Frequencies left/right) (Hz) - Suppression Ratio (SR)   (0-100%)   - EMG (0-100%) - Artifact (ARTF) (0-100%) | - **Response Entropy (RE)**   **numeric values 0-100**   - **State Entropy (SE)**   **numeric values 0-91**   - Burst Suppression Ratio (BSR)   (0-100%) | - **15 EEG stages**   **(A, B0, B1, B2, C0, C1, C2, D0, D1, D2, E0, E1, E2, F0, F1)**   - **Narcotrend Index (NI) numeric values 0-100** - EEG waveform (1 frontal waveform) - Density Spectral Array (DSA): spectrograms representing the power of the EEG frequencies. - Spectral Edge Frequency (SEF) (Hz) - Median Frequency (MF) (Hz) - Burst suppression ratio (BSR) (0-100%) - EMG index (0-100%) | |
| **Moderate to deep sedative range** | **BIS™ 60-40** | **PSI™ 50-25** | **RE 60-40**  **SE 60-40** | **Stages:**   - **Awake (A-B0)** - **Sedated ( B1-B2)** - **Light anaesthesia (C0, C1,C2)** - **General anaesthesia (D0, D1, D2)** - **General anaesthesia with deep hypnosis (E0, E1, E2)** - **General anaesthesia with increasing burst suppression (F0, F1)** | **NI:**  **100-90**  **89-80**  **79-65**  **64-37**  **36-13**  **12-1** |
| ***Comments*** | - Frontal 2-channel (unilateral) and frontal 4-channel (bilateral) monitors are available. - Standalone and modular devices | - Frontal 4-channel monitors - SedLine® can be used simultaneously with O3 Regional Oximetry, Total Hemoglobin (SpHb®), and the Masimo LiDCO® Hemodynamic Monitoring System - Standalone and modular devices | - Frontal electromyograph (FEMG) signals enable a fast response time for RE but do not influence SE. - GE HealthCare Entropy™ Module only (no standalone monitor) | - Frontal 1-channel or 2-channel recording - Standalone and modular devices | |

Table 2. The main features of the most commonly available processed EEG sedation monitors.

## References to supplementary material 2.
